# The landscape of epilepsy-related GATOR1 variants

**DOI:** 10.1038/s41436-018-0060-2

**Published:** 2018-08-10

**Authors:** Sara Baldassari, Fabienne Picard, Nienke E. Verbeek, Marjan van Kempen, Eva H. Brilstra, Gaetan Lesca, Valerio Conti, Renzo Guerrini, Francesca Bisulli, Laura Licchetta, Tommaso Pippucci, Paolo Tinuper, Edouard Hirsch, Anne de Saint Martin, Jamel Chelly, Gabrielle Rudolf, Mathilde Chipaux, Sarah Ferrand-Sorbets, Georg Dorfmüller, Sanjay Sisodiya, Simona Balestrini, Natasha Schoeler, Laura Hernandez-Hernandez, S. Krithika, Renske Oegema, Eveline Hagebeuk, Boudewijn Gunning, Charles Deckers, Bianca Berghuis, Ilse Wegner, Erik Niks, Floor E. Jansen, Kees Braun, Daniëlle de Jong, Guido Rubboli, Inga Talvik, Valentin Sander, Peter Uldall, Marie-Line Jacquemont, Caroline Nava, Eric Leguern, Sophie Julia, Antonio Gambardella, Giuseppe d’Orsi, Giovanni Crichiutti, Laurence Faivre, Veronique Darmency, Barbora Benova, Pavel Krsek, Arnaud Biraben, Anne-Sophie Lebre, Mélanie Jennesson, Shifteh Sattar, Cécile Marchal, Douglas R Nordli, Kristin Lindstrom, Pasquale Striano, Lysa Boissé Lomax, Courtney Kiss, Fabrice Bartolomei, Anne Fabienne Lepine, An-Sofie Schoonjans, Katrien Stouffs, Anna Jansen, Eleni Panagiotakaki, Brigitte Ricard-Mousnier, Julien Thevenon, Julitta de Bellescize, Hélène Catenoix, Thomas Dorn, Martin Zenker, Karen Müller-Schlüter, Christian Brandt, Ilona Krey, Tilman Polster, Markus Wolff, Meral Balci, Kevin Rostasy, Guillaume Achaz, Pia Zacher, Thomas Becher, Thomas Cloppenborg, Christopher J. Yuskaitis, Sarah Weckhuysen, Annapurna Poduri, Johannes R. Lemke, Rikke S. Møller, Stéphanie Baulac

**Affiliations:** 1 Sorbonne Université, UPMC Univ Paris 06, UMR S 1127, F-75013 Paris, France; 2 INSERM, U1127, F-75013 Paris, France; 3 CNRS, UMR 7225, F-75013 Paris, France; 4 Institut du Cerveau et de la Moelle épinière (ICM), Hôpital Pitié-Salpêtrière, F-75013 Paris, France; 5 Department of Genetics, Assistance Publique des Hôpitaux de Paris (AP-HP), Hôpital Pitié-Salpêtrière, F-75013 Paris, France; 6 Department of Clinical Neurosciences, University Hospitals and Medical School of Geneva, Geneva, Switzerland; 7 Department of Genetics, University Medical Center Utrecht, Utrecht, The Netherlands; 8 Service de Génétique, Hospices Civils de Lyon – GHE; CNRS UMR 5292, INSERM U1028, CNRL, et Université Claude Bernard Lyon 1, GHE, Lyon, France; 9 Pediatric Neurology, Neurogenetics, and Neurobiology Unit and Laboratories, A. Meyer Children’s Hospital, Florence, Italy; 10 IRCCS, Istituto delle Scienze Neurologiche of Bologna; Department of Biomedical and Neuromotor Sciences, University of Bologna, Bologna, Italy; 11 Medical Genetics Unit, Polyclinic Sant’ Orsola-Malpighi University Hospital, Bologna, Italy; 12 Department of Neurology-centre de référence des épilepsies rares, University Hospital of Strasbourg, Strasbourg, France; 13 Department of Pediatrics - centre de référence des épilepsies rares, University Hospital of Strasbourg, Strasbourg, France; 14 IGBMC, INSERM, CNRS, Strasbourg University, Strasbourg, France; 15 Department of Pediatric Neurosurgery, Fondation Rothschild, F-75019 Paris, France; 16 Department of Clinical and Experimental Epilepsy, UCL Institute of Neurology, WC1N 3BG, and Chalfont Centre for Epilepsy, Bucks, UK; 17 Stichting Epilepsie Instellingen Nederland, Zwolle/Heemstede, The Netherlands; 18 Leiden University Medical Center, Leiden, The Netherlands; 19 Department of Child Neurology, Brain Center Rudolf Magnus, University Medical Center, Utrecht, The Netherlands; 20 Department of Neurology, Academic Center for Epileptology Kempenhaeghe, Heeze, The Netherlands; 21 Danish Epilepsy Centre, Dianalund, University of Copenhagen, Copenhagen, Denmark; 22 Department of Neurology and Rehabilitation, Tallinn Children’s Hospital, Tallinn, Estonia; 23 Danish Epilepsy Centre, Dianalund, Denmark; 24 Unit of Medical Genetics, CHU La Réunion, Saint Pierre, F-97448 France; 25 Service de Génétique Médicale, Pavillon Lefebvre, Hôpital Purpan CHU Toulouse, Toulouse, France; 26 Institute of Neurology, Department of Medical and Surgical Sciences, University Magna Græcia, Catanzaro, Italy; 27 Epilepsy Center, Clinic of Nervous System Diseases, University of Foggia, Riuniti Hospital, Foggia, Italy; 28 Department of Pediatrics, Institute of Medicine, University Hospital of Udine, Udine, Italy; 29 Centre de Référence Anomalies du Développement et Syndromes Malformatifs et FHU TRANSLAD, CHU de Dijon et Université de Bourgogne, Dijon, France; 30 Service de neurophysiologie et pédiatrie 1, CHU de Dijon, Dijon, France; 31 Department of Paediatric Neurology, Motol University Hospital, 2nd faculty of medicine Charles University, Prague, Czech Republic; 32 Centre Hospitalier Universitaire de Rennes, F-35000 Rennes, France; 33 CHU Reims, Hôpital Maison Blanche, Pôle de Biologie, Service de Génétique, Reims, F-51092 France; 34 CHU Reims, American Memorial Hospital, Service de Pédiatrie, REIMS, F-51092 France; 35 Department of Pediatric Neurology, Rady Children’s Hospital/University of California, San Diego, California USA; 36 Service d’Epileptologie Clinique, CHU de Bordeaux, France; 37 Children’s Hospital Los Angeles, Keck School of Medicine, University of Southern California, Los Angeles, California USA; 38 Division of Genetics and Metabolism, Phoenix Children’s Hospital, Phoenix, Arizona USA; 39 Pediatric Neurology and Muscular Diseases Unit, Department of Neurosciences, Rehabilitation, Ophthalmology, Genetics, Maternal and Child Health, University of Genoa, “G. Gaslini” Institute, Genova, Italy; 40 Department of Medicine, Divisions of Neurology and Respirology, Queen’s University, Kingston, Ontario Canada; 41 Kingston Health Sciences Centre, Kingston, Ontario K7L 2V7 Canada; 42 Pediatric Neurology Department, Timone Hospital, APHM, Marseille, France; 43 Department of Pediatric Neurology, Antwerp University Hospital, Edegem, Belgium; 44 Vrije Universiteit Brussel (VUB), Universitair Ziekenhuis Brussel (UZ Brussel), Neurogenetics Research Group, Laarbeeklaan 101, 1090 Brussels, Belgium; 45 Paediatric Clinical Epileptology, Sleep disorders and Functional Neurology, University Hospitals of Lyon (HCL), Lyon, France; 46 Unité d’épileptologie, Service de Neurologie, CHU, 49033 Angers, France; 47 Inserm UMR 1231 GAD Team, Genetics of Developmental Anomalies, et FHU-TRANSLAD, CHU/Université de Bourgogne-Franche Comté, Dijon, France; 48 Clinique Bernoise, Crans-, Montana, Switzerland; 49 Institute of Human Genetics, University Hospital, Magdeburg, Germany; 50 Epilepsy Center for Children, Brandenburg Medical School, University Hospital, Neuruppin, Germany; 51 Bethel Epilepsy Centre, Bielefeld, Germany; 52 Institute of Human Genetics, University of Leipzig Hospitals and Clinics, Leipzig, Germany; 53 Department of Pediatric Neurology and Developmental Medicine, University Children’s Hospital, Tübingen, Germany; 54 Department of Pediatric Neurology, Children’s Hospital Datteln, Witten/Herdecke University, Datteln, Germany; 55 Institut de Systématique, Evolution, Biodiversité, ISYEB, UMR 7205 CNRS MNHN UPMC EPHE, Paris, France; 56 The Saxon Epilepsy Center Kleinwachau, Radeberg, Germany; 57 Kinderneurologisches Zentrum, Düsseldorf-Gerresheim, Sana Kliniken, Düsseldorf, Germany; 58 Department of Neurology, F.M. Kirby Neurobiology Center, Boston Children’s Hospital, Boston, Massachusetts USA; 59 Division of Epilepsy and Clinical Neurophysiology and Epilepsy Genetics Program, Boston Children’s Hospital, Boston, Massachusetts USA; 60 Department of Neurology, Harvard Medical School, Boston, Massachusetts USA; 61 Neurogenetics Group, VIB-Department of Molecular Genetics, University of Antwerp, Antwerp, Belgium; 62 Danish Epilepsy Centre, Dianalund; Institute for Regional Health research, University of Southern Denmark, Odense, Denmark

**Keywords:** *DEPDC5*, mTORC1 pathway, Genetic focal epilepsy, Focal cortical dysplasia, SUDEP

## Abstract

**Purpose:**

To define the phenotypic and mutational spectrum of epilepsies related to *DEPDC5*, *NPRL2* and *NPRL3* genes encoding the GATOR1 complex, a negative regulator of the mTORC1 pathway

**Methods:**

We analyzed clinical and genetic data of 73 novel probands (familial and sporadic) with epilepsy-related variants in GATOR1-encoding genes and proposed new guidelines for clinical interpretation of GATOR1 variants.

**Results:**

The GATOR1 seizure phenotype consisted mostly in focal seizures (e.g., hypermotor or frontal lobe seizures in 50%), with a mean age at onset of 4.4 years, often sleep-related and drug-resistant (54%), and associated with focal cortical dysplasia (20%). Infantile spasms were reported in 10% of the probands. Sudden unexpected death in epilepsy (SUDEP) occurred in 10% of the families. Novel classification framework of all 140 epilepsy-related GATOR1 variants (including the variants of this study) revealed that 68% are loss-of-function pathogenic, 14% are likely pathogenic, 15% are variants of uncertain significance and 3% are likely benign.

**Conclusion:**

Our data emphasize the increasingly important role of GATOR1 genes in the pathogenesis of focal epilepsies (>180 probands to date). The GATOR1 phenotypic spectrum ranges from sporadic early-onset epilepsies with cognitive impairment comorbidities to familial focal epilepsies, and SUDEP.

## Introduction

Genes encoding components of the amino acid–sensitive branch of the mechanistic target of rapamycin (mTOR) signaling pathway were first implicated in familial focal epilepsies in 2013.^1,2^ DEPDC5 (DEP domain containing protein 5), together with NPRL2 and NPRL3 (nitrogen permease regulator-like 2 and 3) form the GATOR1 (GAP activity towards rags complex 1) complex, a repressor of the mechanistic target of rapamycin complex 1 (mTORC1) pathway.^3^ Heterozygous germline variants in the GATOR1-encoding genes (*DEPDC5*, *NPRL2*, and *NPRL3*) are found in up to 37% of familial focal epilepsies,^4^ as well as in some cases with Rolandic epilepsy^5^ or infantile spasms.^6^ Mendelian focal epilepsies caused by variants in GATOR1 genes encompass familial focal epilepsy with variable foci (FFEVF, MIM 604364),^1,2^ autosomal dominant nocturnal frontal lobe epilepsy (ADNFLE, recently renamed sleep-related hypermotor epilepsy [SHE]),^7^ and familial temporal lobe epilepsy.^2^ It is noteworthy that malformations of cortical development, such as focal cortical dysplasia (FCD), occur in some individuals.^8–13^ FCD type II, which is characterized by dysmorphic neurons and balloon cells, is the most common cause of pediatric drug-resistant epilepsies and represents 9% of the epilepsy surgery population.^14^

In this study, we report clinical and molecular genetic data of a new series of 73 probands with rare variants in *DEPDC5*, *NPRL2*, or *NPRL3* genes, the largest reported so far. We further delineate the GATOR1 phenotype spectrum and provide an updated and critical review of all novel and previously reported GATOR1 epilepsy-related variants, with new guidance for their clinical interpretation.

## Materials and methods

### Patients with GATOR1 variants

Individuals with *DEPDC5*, *NPRL2*, or *NPRL3* variants (missense, splice-site/region altering, nonsense, in-frame/frameshift indels, or copy-number variants [CNVs]) were referred from diagnostic epilepsy centers in Belgium, Canada, Denmark, France, Germany, Italy, Switzerland, the Czech Republic, the Netherlands, the United Kingdom and the United States. Genomic DNA was extracted from peripheral blood lymphocytes. Sanger sequencing was used to confirm single-nucleotide variants, and quantitative polymerase chain reaction (PCR) to confirm CNVs. All probands and affected family members underwent detailed clinical examination, including review of the medical files, magnetic resonance imaging (MRI), and electroencephalographic (EEG) investigations when available. Written informed consent was obtained from all patients included in the study.

### Extraction of variants from the literature

We established a list of all epilepsy-related GATOR1 variants reported in 24 original publications from PubMed (https://www.ncbi.nlm.nih.gov/pubmed; accessed September 2017; Table S2 and Supplementary Information).

### Variant annotation and classification

We annotated novel and previously reported variants based on the longest transcripts of *DEPDC5* (Refseq NM_001242896; NP_001229825), *NPRL2* (Refseq NM_006545; NP_006536) and *NPRL3* (Refseq NM_001077350; NP_001070818), using the online version of Variant Effect Predictor (VEP) for human GRCh37 (http://grch37.ensembl.org/Homo_sapiens/Tools/VEP).

Population allele frequencies for each variant were extracted from the Genome Aggregation Database browser (gnomAD, http://gnomad.broadinstitute.org/; accessed in July 2017).^15^

We used in silico prediction tools to assess the pathogenicity of variants: M-CAP (Mendelian Clinically Applicable Pathogenicity) for missense variants;^16^ and HSF (Human Splice Finder v3.0) for splice-region variants.

### Comparison of GATOR1 variants in epilepsy and gnomAD cohorts

Coding and splice altering variants in *DEPDC5*, *NPRL2*, and *NPRL3* were extracted from the gnomAD database and annotated using VEP on the transcript of interest. The distribution of the different types of variants (missense, splice-site/region altering, nonsense, in-frame/frameshift indels, and CNVs) and the proportion of M-CAP predicted deleterious rare missense variants in the gnomAD cohort were then compared with those found in epilepsy subjects: 110 previously published and 73 from the present cohort.

## Results

### Clinical features in the new patient cohort

We assembled a cohort of 73 previously unreported probands with rare variants in *DEPDC5*, *NPRL2*, and *NPRL3* through international networking with diagnostic epilepsy centers. The main clinical features of the 73 probands are presented in Table 1; the complete clinical description of the 73 probands (and 26 affected relatives) is presented in Table S1. All percentages are related to the total number of probands that could be evaluated for a given feature.

**Table 1:** Demographics and clinical features of the 73 probands reported in this study

	**GATOR1**	***DEPDC5***	***NPRL2***	***NPRL3***
**Probands**	73	63	3	7
**Gender (male:female)**	36:37	33:30	1:2	2:5
**Epilepsy family history**	40/73 (55%)	33/63	1/3	6/7
**Inheritance**				
De novo	2/47 (4%)	2/40	0/1	0/6
Inherited	45/47 (96%)	38/40	1/1	6/6
**Age at sz onset (years)**	4.4 (0–16)	4.4 (0–16)	1.5 (0–3.7)	5.7 (1–16)
**Epilepsy phenotype**				
Sleep-related hypermotor epilepsy	26/73 (36%)	22/63	1/3	3/7
Frontal lobe epilepsy	12/73 (16%)	9/63	1/3	2/7
Temporal lobe epilepsy	1/73 (1%)	1/63	0/3	0/7
Focal epilepsy (unspecified)	23/73 (32%)	21/63	0/3	2/7
Infantile spasms	7/73 (10%)	6/63	1/3	0/7
Generalized epilepsy	3/73 (4%)	3/63	0/3	0/7
Focal febrile seizures	1/73 (1%)	2/63	0/3	0/7
**Cognitive comorbidities**				
Normal	37/68 (54%)	30/59	0/2	7/7
Language delay	10/68 (15%)	9/59	0/2	1/7
Intellectual disability	18/68 (26%)	16/59	2/2	0/7
Other deficits	7/68 (9%)	7/59	0/2	0/7
**Psychiatric features**				
None	37/65 (57%)	30/56	2/2	5/7
ADHD / attention deficit	10/65 (15%)	10/56	0/2	0/7
ASD / autistic features	6/65 (9%)	6/56	0/2	0/7
Oppositional disorder	12/65 (18%)	11/56	0/2	1/7
Anxiety and/or depression	5/65 (8%)	5/56	0/2	0/7
Others	5/65 (8%)	4/56	0/2	1/7
**Neuroimaging**				
Normal	44/71 (62%)	37/61	1/3	6/7
Brain abnormality	27/71 (38%)	24/61	2/3	1/7
Malformations of cortical development	17/27 (63%)	15/24	2/2	0/1
Focal cortical dysplasia	14/27 (52%)	12/24	2/2	0/1
Hemimegalencephaly	1/27 (4%)	1/24	0/2	0/1
Subcortical heterotopia	1/27 (4%)	1/24	0/2	0/1
Hemispheric cortical dysplasia	1/27 (4%)	1/24	0/2	0/1
Hippocampal sclerosis/atrophy	4/27 (15%)	4/24	0/2	0/1
**Antiepileptics**				
Monotherapy	8/72 (11%)	7/62	0/3	1/7
2 Antiepileptic drugs	15/72 (21%)	13/62	0/3	2/7
≥3 Antiepileptic drugs	49/72 (68%)	42/62	3/3	4/7
Good outcome	33/71 (46%)	29/61	0/3	4/7
Drug-resistant	38/71 (54%)	32/61	3/3	3/7
**Surgery**	11/73 (15%)	9/73	2/3	0/7
Surgery outcome				
Engel I	6/10 (60%)	5/8	1/2	0/0
Engel II	2/10 (20%)	2/8	0/2	0/0
Engel III	1/10 (10%)	0/8	1/2	0/0
Engel IV	1/10 (10%)	1/8	0/2	0/0
**SUDEP** ^a^	9/73 (12%)	8/63	0/3	1/7
**Cancer**	2/73 (3%)	1/63	0/3	1/7

*Sz* seizures, *ADHD* attention-deficit/hyperactivity disorder, *ASD* autism spectrum disorder, *Engel I* free of disabling seizures, *Engel II* rare disabling seizures, *Engel III* worthwhile improvement, *Engel IV* no worthwhile improvement

^a^SUDEP sudden unexpected death in epilepsy cases refer to probands or affected family members

All except two cases (screened for research purposes) were referred for epilepsy genetic diagnosis. A known family history of epilepsy was reported in 55% of the probands. The age at epilepsy onset ranged from the first days of life to 16 years (mean: 4.4 years). Thirty percent (22/73) of the probands had early-onset epilepsy (≤1 year), among which focal seizures were reported in 68% (15/22), infantile spasms in 27% (6/22), or a combination of generalized and focal seizures in one proband. In the cohort, the seizure spectrum comprised 36% (26/73) of sleep-related hypermotor seizures, 16% (12/73) of frontal lobe seizures, 32% (23/73) of focal seizures with undetermined origin, and 10% of infantile spasms. Seizures occurred predominantly during sleep in 48% (35/73) of the probands. Interictal epileptiform abnormalities (sharp waves, spikes or spike-and-waves, focal or multifocal) were detected by EEG or stereoelectroencephalography (SEEG) in 83% (55/66) of the probands, and in up to 90% (18/20) among those affected with SHE. Six other probands (9%) had interictal focal slow waves, while a normal interictal EEG was reported in only five probands (7.5%).

Patients were followed up for a period ranging from 8 months to 2 years (for 6 young probands) up to 58 years (>2 years for 67 probands). Seizure outcome was variable, with a rate of resistance to antiepileptic drugs (AEDs) of 54% (38/71), and up to 65% among probands with SHE. Only 11% of probands were seizure-free on the first AED treatment, while four AEDs were tested on average. Among the seizure-free patients on monotherapy, 5/8 received sodium channel–blocking AEDs, which are classical first-line drugs to treat focal epilepsies. Five other probands were treated with a ketogenic diet, in addition to AEDs, with a seizure improvement in two of them.

Neuroimaging investigations, including MRI and fluorodeoxyglucose-positron emission tomography (FDG-PET), revealed abnormalities in 38% (27/71) of the probands, among which 24% (17/71) were malformations of cortical development. Focal cortical dysplasia (FCD) was diagnosed or suspected in 20% (14/71) of the probands, a hemispheric cortical dysplasia in 1 proband, hemimegalencephaly in 1 proband, and focal subcortical heterotopia in another proband. Four additional probands showed hippocampal atrophy/sclerosis, and four others had nonspecific brain abnormalities at MRI (Table S1). FDG-PET showed a focal hypometabolism with a normal MRI, suggestive of FCD, in two additional patients.

Epilepsy resective surgery was performed in 11 probands, including 2 with a normal MRI, and 9 with MRI suggestive of either FCD (8 probands) or hemimegalencephaly (1 proband). According to the Engel Epilepsy Surgery Outcome Scale, 60% (6/10) of probands have been seizure-free since surgery (Engel I score, follow-up range: 9 months to 5 years), while 20% (2/10) were almost seizure-free (Engel II score, follow-up: 10 months and 15 months), 1 had a worthwhile improvement (Engel III score, follow-up: 7 years), and 1 had no worthwhile improvement (Engel IV score, follow-up: 7 years). Histological examinations confirmed FCD type II in six probands (five FCD IIa, one FCD IIb) and FCD type I in two probands.

Cognitive impairment and/or psychiatric comorbidities were reported in 60% of the probands of this cohort, and in 76% (16/21) if considering only probands with age at onset ≤1 year. Neurocognitive deficits were reported in 44% (30/68) of the probands. Mild or moderate intellectual disability was reported in 20% (14/68) and severe in 6% (4/68); language learning delay was reported in 15% (10/68) and 9% (7/68) had other cognitive disturbances. In the subgroup of probands with age at onset of epilepsy ≤1 year, 20% (4/19) had a severe intellectual disability or global developmental delay, 8 had a mild or moderate intellectual disability, and 7 had normal cognitive function. Psychiatric comorbidities were diagnosed in 43% (28/65) of the probands: oppositional disorder in 12 probands, attention-deficit hyperactivity disorder (ADHD) or attention deficit in 10, autism spectrum disorder (ASD) diagnosis or autistic features in 6, anxiety and/or depression in 5, disinhibited social engagement disorder in 1, and obsessive-compulsive disorder in 1.

Because somatic variants in GATOR1-encoding genes (>400 in *DEPDC5*, >80 in *NPRL2*, and >100 in *NPRL*3 in the COSMIC database) have been mentioned in various cancers,^3,17^ we examined cancer occurrence in epilepsy probands carrying germline GATOR1 variants. Two probands developed cancer, one developed Hodgkin lymphoma, and one developed breast cancer. We could not conclude about a link between GATOR1 germline variants and increased risk of cancer.

In this series of 73 families, nine individuals (one proband and eight affected relatives) succumbed to sudden unexpected death in epilepsy (SUDEP). SUDEP is a “non-accidental, non-suicidal and non-drowning death in people with epilepsy, unrelated to a documented status epilepticus, in which postmortem examination does not reveal a structural or toxicologic cause of death.”^18,19^ According to the SUDEP classification,^19^ one proband had a definite SUDEP (confirmed by autopsy, which did not reveal a cause of death) and eight individuals had a probable SUDEP (same definition as “definite SUDEP,” but without autopsy confirmation). Eight SUDEP cases belonged to seven families with multiple affected members in which a pathogenic null variant was identified in *DEPDC5* (two SUDEP cases were in the same family) and one carried a pathogenic loss-of-function (LoF) variant in *NPRL3* segregating in a large autosomal dominant SHE family (pedigrees are shown in Fig. S1). Genomic DNA of 3/9 SUDEP cases, who had a history of sleep-related focal epilepsy, was available for genetic analysis and confirmed that these individuals carried the pathogenic variant (the remaining SUDEP cases could not be tested for segregation of the variants). The mean age at the time of SUDEP was 36.8 years (age range: 19–59 years).

### GATOR1 variants in the new cohort and variant classification framework

Germline heterozygous variants in GATOR1-encoding genes were identified in 73 unrelated probands by different diagnostic targeted gene panels and by exome sequencing for research purposes in two cases. Variants were inherited in 45 cases and occurred de novo in two probands, while information was not available in the remaining 26 cases. Variants were dominantly inherited from an asymptomatic parent in 64% (29/45) of the probands, illustrating the reduced penetrance of GATOR1 variants.

**Fig. 1:**
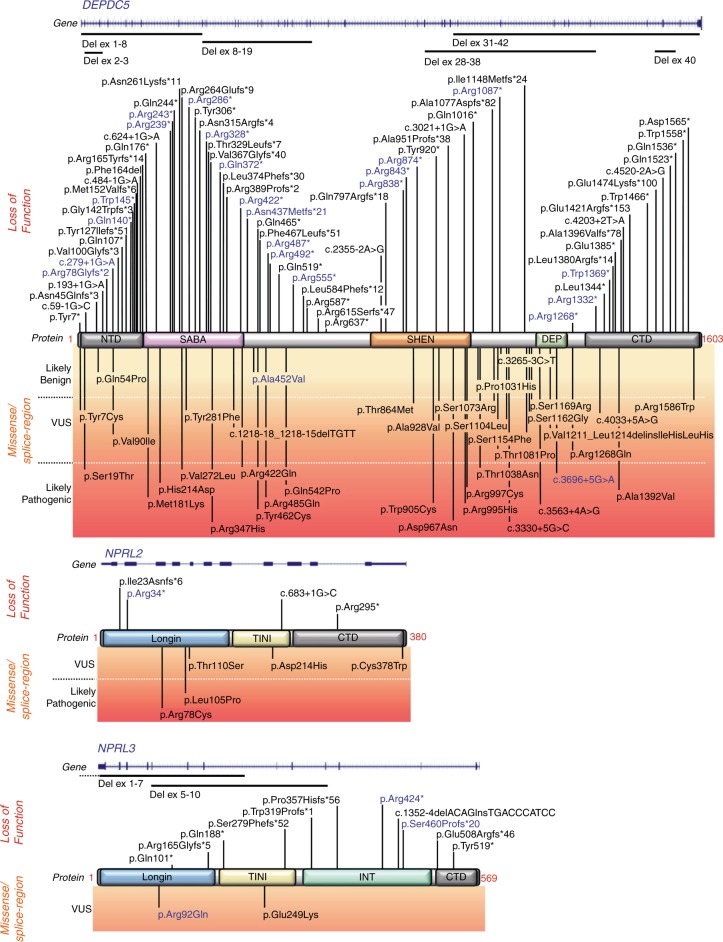
**Schematic diagram showing the domain/structural organization of DEPDC5, NPRL2, and NPRL3 proteins and the positions of the 140 distinct epilepsy-related variants reported so far according to the 3D-structure of the GATOR1 protein complex.** In the upper panels of each protein are indicated loss-of-function (LoF) variants, while missense and splice-region variants are shown in the bottom part and classified according to our novel proposed framework. Recurrent variants are indicated in blue. *VUS* variant of uncertain significance^24^

**Fig. 2:**
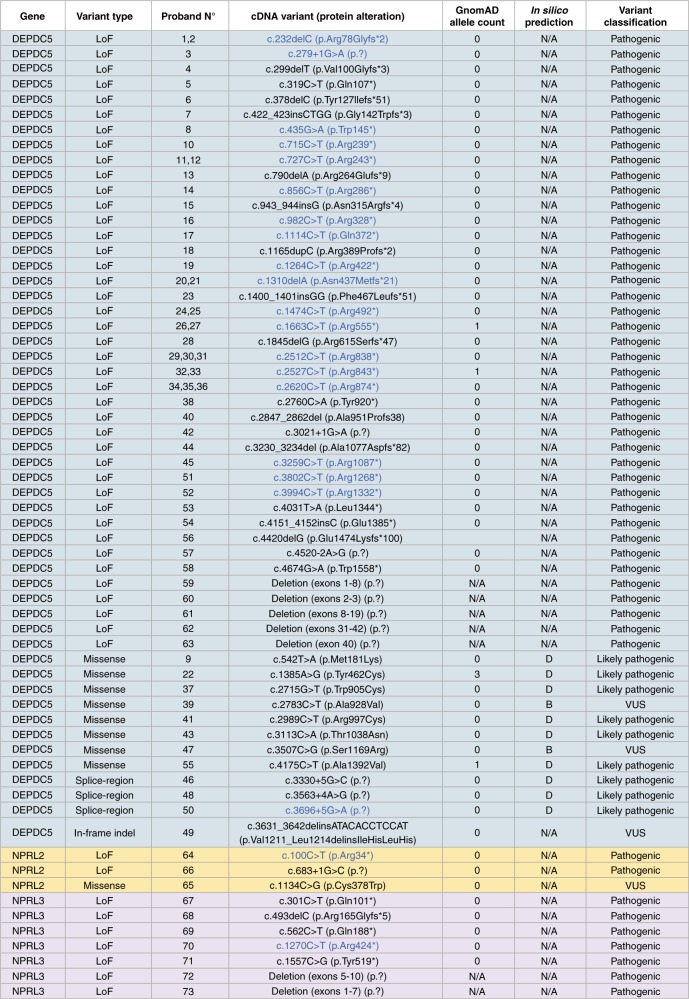
**All 63 distinct GATOR1 variants identified in the new patient cohort**. For missense variants, Mendelian Clinically Applicable Pathogenicity (M-CAP) was used to predict possible pathogenic (D) or possible benign (B) variants. Human Splice Finder (HSF) v3.0 was used to discriminate splice-region variants with a possible impact on the splicing (D) and those not predicted to impact the splicing of mRNA (B). *cDNA* complementary DNA, *VUS* variant of uncertain significance, *LoF* loss of function, *N/A* not available. Recurrent variants are indicated in blue

Overall, 63 distinct variants were identified: 53 in *DEPDC5*, 3 in *NPRL2*, and 7 in *NPRL3* (Figs. 1 and 2). Among these, 46 were novel (including 39 single-nucleotide variants and seven CNVs) and 16 were newly defined as recurrent variants; 34 were LoF variants (nonsense, splice-site, frameshift indels, and CNVs). According to the new American College of Medical Genetics and Genomics (ACMG) guidelines for variant classification of Mendelian disease,^20^ the 34 newly reported LoF variants were classified as pathogenic. Moreover, nonsense-mediated RNA decay (NMD) was previously suggested for *DEPDC5* and *NPRL3* transcripts with nonsense variants, confirming that haploinsufficiency is the pathogenic mechanism in GATOR1-related disorders.^2,7,12,13^

**Fig. 3:**
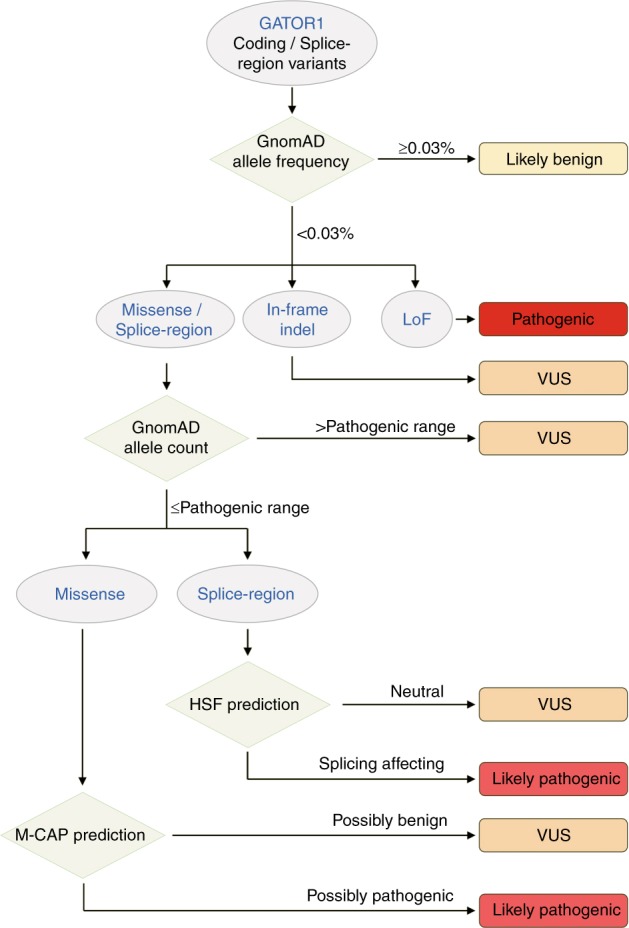
**Classification framework for epilepsy-related GATOR1 variants**. Pathogenic range: 6 alleles for *DEPDC5*, 3 alleles for *NPRL2*, and 4 alleles for *NPRL3*

Next, we asked whether the remaining 12 novel variants (9 missense, 2 splice-region, and 1 in-frame indel) might be deleterious. Because of lack of both functional assays and strong genetic evidence (segregation in more than three affected family members, or recurrence of a given variant in unrelated epilepsy probands), these variants would be classified as variants of uncertain significance (VUS) according to the ACMG framework. However, because they are rare (or absent) in the control population and found in patients with a phenotype compatible with GATOR1-related focal epilepsy, we asked whether such variants might nevertheless be clinically relevant. We therefore propose a new classification framework specifically adapted to GATOR1 genes, based on gnomAD allele frequencies and in silico pathogenicity predictions (Fig. 3). First, we defined an allele frequency threshold for likely benign variants, adjusting the BA1 ACMG rule (which sets the benign classification for variants at an allele frequency ≥5%, which corresponds to 13,863 alleles in gnomAD)^21^ based on the prevalence of GATOR1-related epilepsies, as recently proposed for *MYH7*-associated cardiomyopathies.^22^ We estimated the prevalence of genetic focal epilepsies in the population to be 0.32%, a contribution of *DEPDC5* gene to genetic focal epilepsy of 9.4%^23^ and a penetrance of 60%^4^ and then calculated an allele frequency threshold of 0.03% for likely benign variants (Supplementary Information). Therefore, variants with gnomAD allele frequencies above 0.03% (83 alleles) were classified as likely benign. Besides, ACMG rules specify that eight alleles should be considered as the maximum allele count within the “pathogenic range.”^20^ We also adjusted this pathogenic range to reflect the occurrence of LoF variants in GATOR1 genes in the gnomAD control population. Hence, we set the pathogenic range allele count of *DEPDC5*, *NPRL2*, and *NPRL3* to 6, 3, and 4 alleles in gnomAD, corresponding to the maximum allele counts of nonsense and frameshift indels in the gnomAD cohort in each GATOR1 gene. We also used M-CAP, a novel clinical pathogenicity web-based classifier that compiles predictions from nine different pathogenicity prediction tools and conservation scores for missense variants,^16^ and HSF to predict the effect of splice-region variants. Hence, missense and splice-region variants with a gnomAD frequency within the pathogenic range threshold and predicted possibly pathogenic by M-CAP or to affect splicing by HSF were defined as likely pathogenic; otherwise they were considered as VUS. Finally, as the impact of rare in-frame indels cannot be predicted by in silico tools, they were classified as VUS.

In summary, according to this novel classification framework (Fig. 3), six missense and two splice-region variants were classified as likely pathogenic, while the remaining three missense variants and the in-frame indel were classified as VUS (Figs. 1 and 2).

### Reclassification of literature-reported GATOR1 variants

We then reviewed the previously published germline epilepsy-related variants in the three GATOR1 genes. In total, we listed 78 distinct variants in *DEPDC5*, 7 in *NPRL2*, and 9 in *NPRL3*. Among them, 64% (60/94) were LoF and were classified as pathogenic, 31% (29/94) were missense, 4% (4/94) were splice-region variants, and 1 was an in-frame deletion (p.Phe164del) classified as pathogenic because it segregated in three large FFEVF families^1^ and was absent in gnomAD (Fig. 1, Table S2 and Supplementary Information).

We then applied our classification framework to evaluate the clinical relevance of the 33 missense/splice-region variants (Fig. 1 and Table S2). Three missense and one splice-region variants were classified as likely benign because they were found at allele counts of 94 (p.Gln54Pro), 118 (p.Ala452Val), 139 (p.Pro1031His), and 181 (c.3265-3C>T) in gnomAD. Therefore, four previously reported variants are unlikely to be involved in the pathogenesis of epilepsy. In addition, we reclassified 12 variants as likely pathogenic and 17 as VUS. Among the 17 VUS, 7 had already been classified as VUS, while the remaining 10 had been initially classified as (likely) pathogenic, mainly because they were absent or rare in the public databases available at that time (ExAC, EVS, 1000 Genomes). Thanks to the recent high-throughput sequencing and data aggregation gnomAD initiative, which includes 123,136 exome sequences and 15,496 genome sequences, it now appears that most of these variants have a frequency above the pathogenic range, and consequently were reclassified as VUS. Three variants, rare or absent in gnomAD, were reclassified as VUS because they were predicted benign by M-CAP or HSF.

### Global GATOR1 mutational spectrum

**Fig. 4:**
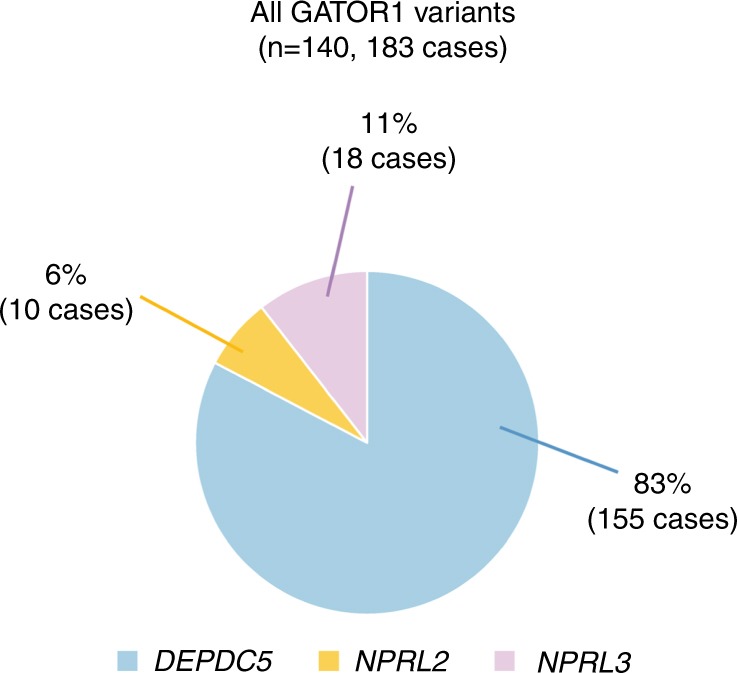
**Distribution of the 140 distinct epilepsy-related GATOR1 variants among the 183 probands reported so far**. Percentages and number of probands are indicated for each gene

Overall, 140 distinct GATOR1 variants have been described in 183 epilepsy probands so far. Most variants are LoF (67%), followed by missense (27%), splice-region variants (4%), and in-frame indels (1%). Recurrent GATOR1 pathogenic LoF variants (*n* = 24) were reported in unrelated cases, indicating mutational hotspots or founder effects (represented in blue in Fig. 1 and Table S2). Among the GATOR1 genes, *DEPDC5* is the most frequently mutated gene accounting for 85% (155/183) of all cases (Fig. 4). This may be explained by the greater length of *DEPDC5* transcript (5551 bp) compared with *NPRL2* (1700 bp) and *NPRL3* (2881 bp), and the fact that *NPRL2* and *NPRL3* have been tested in a lower number of individuals due to their more recent discovery.

We then compared the type of variants among the 140 epilepsy-related GATOR1 variants with those listed in gnomAD. Excluding silent variants, the distribution of GATOR1 variants in gnomAD was 80% missense, 15% splice-region variants, 4% LoF, 1% in-frame indels, and was similar for all three GATOR1 genes (Fig. S2). This contrasts with the epilepsy cohort, in which a predominance of LoF variants is observed (67% as a mean among all GATOR1 genes, Fig. S2). As expected, this enrichment of LoF variants in the epilepsy patient’s cohort is consistent with the presumed role of these variants in the pathogenesis of epilepsy. Nonetheless, the presence of rare LoF variants in gnomAD is likely to reflect the reduced penetrance and milder presentations observed in GATOR1 families.^4^ As expected, the percentage of individuals with likely pathogenic missense variants in *DEPDC5* and *NPRL2* genes was significantly higher in the epilepsy cohort compared with the gnomAD cohort (47% vs. 1.7% for *DEPDC5* [*p* value <2.2e^−16^], and 40% vs. 4.7% for *NPRL2* [*p* value = 0.02], two-tailed Fisher’s exact test). This result confirms that the hereby proposed classification method is a valuable resource to help discriminate likely pathogenic and likely benign variants identified in GATOR1 genes.

The architecture of GATOR1 protein complex was recently solved by cryo-electron microscopy, revealing five protein domains in DEPDC5: N-terminal domain (NTD), structural axis for binding arrangement (SABA) domain, steric hindrance for enhancement of nucleotidase activity (SHEN) domain, DEP domain, and C-terminal domain (CTD).^24^ We analyzed the distribution of the 31 missense variants along the different DEPDC5 domains but did not find any significant clustering (Fig. S3, Supplementary Information).

## Discussion

With over 180 unrelated families described to date, genes of the GATOR1-mTORC1 pathway (*DEPDC5*, *NPRL2*, and *NPRL3*) are collectively the most frequently mutated genes in focal epilepsies, among which *DEPDC5* is predominantly found (85% of all cases).

We reviewed the clinical features of all 183 GATOR1 probands reported so far (in the literature and in this study). As the phenotype is similar between individuals carrying variants in *DEPDC5*, *NPRL2* or *NPRL3* (Supplementary Information, Table S2), we define a “GATOR1” phenotype. The GATOR1-related epilepsy phenotype consists mostly in SHE (characterized by sleep-related focal hypermotor seizures), accounting for 25% (45/183) of all cases; other focal epilepsies (including frontal, temporal, frontotemporal, occipital, parietal, centrotemporal epilepsies) have been described in 54% (93/172) of the cases, while FFEVF was diagnosed in 9% (16/183). Infantile spasms were also part of the GATOR1 phenotype spectrum, occurring in 6.6% (12/183) of all reported GATOR1-mutated patients (including the study by Carvill et al.^6^). Drug resistance in this novel series was observed in half of the probands, and up to 65% in probands with SHE, supporting previous studies based on smaller cohorts of families with SHE/ADNFLE (7/9 drug-resistant individuals)^7^ or infantile spasms (4/5)^6^. Malformations of cortical development (mostly FCD) were reported in 24% of the probands. A good surgery outcome (Engel scores I–II) was achieved in 80% of them. Previous studies also reported favorable surgery outcome in FCD cases with variants in GATOR1 genes, with seizure remission in six individuals and a worthwhile improvement in three others,^9,12,13^ indicating that the presence of a germline GATOR1 variant does not represent a contraindication for surgery. In one patient with an inherited *DEPDC5* variant who underwent epilepsy surgery (proband 14), a somatic second-hit variant, in *trans* configuration, was detected in the DNA extracted from the resected epileptogenic zone, suggesting that a loss-of-function biallelic mutational mechanism in a negative regulator of mTORC1 causes FCD.^25^ In this study, cognitive impairment and/or psychiatric comorbidities were commonly reported (60% of the probands), although severe cognitive impairment occurred only in early-onset cases, mostly presenting with infantile epileptic spasms. Previous studies also mentioned neuropsychiatric comorbidities in ~36% of the probands with GATOR1 variants (Table S2). The higher percentage reported in this study may reflect the inclusion of a greater number of individuals (30%) with early-onset epilepsy (≤1 year), which is classically associated with intellectual disability,^26^ and may therefore reflect a bias due to the fact that younger and more severely affected patients are more often referred for molecular diagnosis. Moreover, ASD was diagnosed in 9% of the probands of this series, confirming a recent study reporting a *DEPDC5* variant (p.Tyr1546His) in a patient with ASD and frontal epileptiform discharges but no clinical seizures.^27^

Here, we reported nine SUDEP cases among the 73 new families; two were part of the same family. Two previous studies have described SUDEP cases among three families with inherited *DEPDC5*, *NPRL2*, or *NPRL3* variants.^13,28^ Moreover, in a retrospective SUDEP cohort, *DEPDC5* variants were found in ~10% (6/61) of cases.^29^ Overall, SUDEP cases were reported in 17 families over 183 (9.3%), emphasizing a possible increased risk of SUDEP in individuals with GATOR1 variants when compared with the global incidence of SUDEP of 0.22/1000 individuals/year in children and 1.2/1000 individuals/year in adults with epilepsy.^30^ The early age at onset of epilepsy, drug resistance, and predominant sleep-related occurrence of seizures observed in GATOR1-mutated patients, are well-recognized SUDEP risk factors.^18,19^ However, like *SCN1A* variants in Dravet syndrome, GATOR1 variants might confer a higher risk for SUDEP by themselves, although the GATOR1-related physiopathological mechanisms of SUDEP remain unknown so far.^31^ Assessing whether GATOR1 variants increase SUDEP risk via direct effects on cardiorespiratory functions may have profound clinical impact.

Finally, we attempted to identify genotype–phenotype correlations among the 183 cases and asked whether pathogenic LoF variants might cause a more severe phenotype with FCD, SUDEP, or infantile spasms than likely pathogenic/VUS variants. We reviewed the literature and this study, and found that 28/32 patients with FCD, 15/17 SUDEP families, and 9/11 with infantile spasms had a pathogenic LoF variant (Table S2). We did not disclose any statistically significant genotype–phenotype correlation.

The assignment of pathogenicity of LoF variants (representing 67% of all epilepsy-related GATOR1 variants) is straightforward because haploinsufficiency has been shown to be the pathogenic mechanism.^2,7,12,13^ However, the puzzling and unanswered question is whether the 31% of rare missense or splice-region variants have a clinical significance. Several obstacles are: (1) the lack of functional evidence to prove deleterious effect on protein function; (2) the lack of a strong segregation support, and incomplete penetrance (with variants inherited from asymptomatic parents in 64% of the cases); and (3) the absence of recurrent missense variants (except for the p.Arg92Gln variant in *NPRL3*) as shown for LoF variants (suggesting mutational hotspots). To respond to an important need for diagnostic testing, we adapted the ACMG classification to GATOR1-related epilepsies, to provide an updated variant classification framework for clinical interpretation. Considering that the frequency of a given variant in the general population is to date the most reliable criterion for interpreting its clinical significance, we took advantage of the recent release of gnomAD to classify all epilepsy-related GATOR1 variants using a threshold specifically dedicated to GATOR1-related epilepsies. Our classification framework together with an accurate delineation of the GATOR1 phenotype spectrum will help clinicians and geneticists for the clinical interpretation of GATOR1 variants. While none of the missense/splice-region variants was classified as pathogenic according to our framework, future in vitro functional assays aimed to measure mTORC1 activity should allow to definitively settle on their pathogenicity.

To conclude, this collaborative study emphasizes that GATOR1 genes of the amino acid–sensing branch of the mTORC1 pathway, especially *DEPDC5*, are key contributors to a broad spectrum of lesional and nonlesional epilepsies, with variable but highly consistent phenotypes. The pathogenic molecular mechanism linked to GATOR1 haploinsufficiency is a hyperactivation of mTORC1 pathway, as shown in human resective brain specimens^12,13^ and rodent models.^32–34^ However, how this signaling cascade alters neuronal network function, neuronal development, and ultimately leads to seizures remains to be elucidated. While a high rate of drug resistance to traditional AEDs is often reported, this study also points out a favorable epilepsy surgery outcome in some cases. For patients who are not eligible for surgery, alternative therapeutic approaches targeting GATOR1/mTORC1 complexes are urgently needed. Currently available mTORC1 inhibitors, such as rapamycin, represent promising drugs in the treatment of focal epilepsies.^35^ Yet, selective agonists of GATOR1 could act as antiepileptogenic drugs as well, which could ultimately lead to reduced side effects and a targeted therapy.

## Electronic supplementary material

Supplementary Figure S1

Supplementary Figure S2

Supplementary Figure S3

Supplementary Information

Supplementary Table S1

Supplementary Table S2

## References

[1] Dibbens LM, de Vries B, Donatello S (2013). Mutations in DEPDC5 cause familial focal epilepsy with variable foci. Nat Genet.

[2] Ishida S, Picard F, Rudolf G (2013). Mutations of DEPDC5 cause autosomal dominant focal epilepsies. Nat Genet.

[3] Bar-Peled L, Chantranupong L, Cherniack AD (2013). A tumor suppressor complex with GAP activity for the Rag GTPases that signal amino acid sufficiency to mTORC1. Science.

[4] Baulac S (2016). mTOR signaling pathway genes in focal epilepsies. Progress Brain Res.

[5] Lal D, Reinthaler EM, Schubert J (2014). DEPDC5 mutations in genetic focal epilepsies of childhood. Ann Neurol.

[6] Carvill GL, Crompton DE, Regan BM (2015). Epileptic spasms are a feature of DEPDC5 mTORopathy. Neurol Genet.

[7] Picard F, Makrythanasis P, Navarro V (2014). DEPDC5 mutations in families presenting as autosomal dominant nocturnal frontal lobe epilepsy. Neurology.

[8] Scheffer IE, Heron SE, Regan BM (2014). Mutations in mammalian target of rapamycin regulator DEPDC5 cause focal epilepsy with brain malformations. Ann Neurol.

[9] Baulac S, Ishida S, Marsan E (2015). Familial focal epilepsy with focal cortical dysplasia due to DEPDC5 mutations. Ann Neurol.

[10] D’Gama AM, Geng Y, Couto JA (2015). Mammalian target of rapamycin pathway mutations cause hemimegalencephaly and focal cortical dysplasia. Ann Neurol.

[11] Scerri T, Riseley JR, Gillies G (2015). Familial cortical dysplasia type IIA caused by a germline mutation in DEPDC5. Ann Clin Transl Neurol.

[12] Sim JC, Scerri T, Fanjul-Fernandez M (2016). Familial cortical dysplasia caused by mutation in the mammalian target of rapamycin regulator NPRL3. Ann Neurol.

[13] Weckhuysen S, Marsan E, Lambrecq V (2016). Involvement of GATOR complex genes in familial focal epilepsies and focal cortical dysplasia. Epilepsia.

[14] Blumcke I, Spreafico R, Haaker G (2017). Histopathological findings in brain tissue obtained during epilepsy surgery. N Engl J Med.

[15] Lek M, Karczewski KJ, Minikel EV (2016). Analysis of protein-coding genetic variation in 60,706 humans. Nature.

[16] Jagadeesh KA, Wenger AM, Berger MJ (2016). M-CAP eliminates a majority of variants of uncertain significance in clinical exomes at high sensitivity. Nat Genet.

[17] Forbes SA, Beare D, Boutselakis H (2017). COSMIC: somatic cancer genetics at high-resolution. Nucleic Acids Res.

[18] Nashef L, So EL, Ryvlin P, Tomson T (2012). Unifying the definitions of sudden unexpected death in epilepsy. Epilepsia.

[19] Devinsky O, Hesdorffer DC, Thurman DJ (2016). Sudden unexpected death in epilepsy: epidemiology, mechanisms, and prevention. Lancet Neurol.

[20] Nykamp K, Anderson M, Powers M (2017). Sherloc: a comprehensive refinement of the ACMG-AMP variant classification criteria. Genet Med.

[21] Richards S, Aziz N, Bale S (2015). Standards and guidelines for the interpretation of sequence variants: a joint consensus recommendation of the American College of Medical Genetics and Genomics and the Association for Molecular Pathology. Genet Med.

[22] Kelly MA, Caleshu C, Morales A (2018). Adaptation and validation of the ACMG/AMP variant classification framework for MYH7-associated inherited cardiomyopathies: recommendations by ClinGen’s Inherited Cardiomyopathy Expert Panel. Genet Med.

[23] Ricos MG, Hodgson BL, Pippucci T (2016). Mutations in the mammalian target of rapamycin pathway regulators NPRL2 and NPRL3 cause focal epilepsy. Ann Neurol.

[24] Shen K, Huang RK, Brignole EJ (2018). Architecture of the human GATOR1 and GATOR1-Rag GTPases complexes. Nature.

[25] Ribierre Théo, Deleuze Charlotte, Bacq Alexandre, Baldassari Sara, Marsan Elise, Chipaux Mathilde, Muraca Giuseppe, Roussel Delphine, Navarro Vincent, Leguern Eric, Miles Richard, Baulac Stéphanie (2018). Second-hit mosaic mutation in mTORC1 repressor DEPDC5 causes focal cortical dysplasia–associated epilepsy. Journal of Clinical Investigation.

[26] Berg AT, Zelko FA, Levy SR (2012). Age at onset of epilepsy, pharmacoresistance, and cognitive outcomes: a prospective cohort study. Neurology.

[27] Burger BJ, Rose S, Bennuri SC (2017). Autistic siblings with novel mutations in two different genes: insight for genetic workups of autistic siblings and connection to mitochondrial dysfunction. Front Pediatr.

[28] Nascimento FA, Borlot F, Cossette P (2015). Two definite cases of sudden unexpected death in epilepsy in a family with a DEPDC5 mutation. Neurol Genet.

[29] Bagnall RD, Crompton DE, Petrovski S (2016). Exome-based analysis of cardiac arrhythmia, respiratory control, and epilepsy genes in sudden unexpected death in epilepsy. Ann Neurol.

[30] Harden C, Tomson T, Gloss D (2017). Practice guideline summary: sudden unexpected death in epilepsy incidence rates and risk factors: Report of the Guideline Development, Dissemination, and Implementation Subcommittee of the American Academy of Neurology and the American Epilepsy Society. Neurology.

[31] Cooper MS, McIntosh A, Crompton DE (2016). Mortality in Dravet syndrome. Epilepsy Res.

[32] Marsan E, Ishida S, Schramm A (2016). Depdc5 knockout rat: a novel model of mTORopathy. Neurobiol Dis.

[33] Yuskaitis CJ, Jones BM, Wolfson RL (2017). A mouse model of DEPDC5-related epilepsy: neuronal loss of Depdc5 causes dysplastic and ectopic neurons, increased mTOR signaling, and seizure susceptibility. Neurobiol Dis.

[34] Hughes J, Dawson R, Tea M (2017). Knockout of the epilepsy gene Depdc5 in mice causes severe embryonic dysmorphology with hyperactivity of mTORC1 signalling. Sci Rep.

[35] Myers KA, Scheffer IE (2017). DEPDC5 as a potential therapeutic target for epilepsy. Expert Opin Ther Targets.

