# Supplementary material for: The landscape of epilepsy-related GATOR1 variants
Source: Genet Med. 2018 Aug 10;21(2):398–408. doi: 10.1038/s41436-018-0060-2 (PMC6292495; doi:10.1038/s41436-018-0060-2)

Supplementary Figure S1: Pedigrees of GATOR1 families with SUDEP reported cases. SHE: sleep-related hypermotor epilepsy; MCD: malformation of cortical development; SUDEP: sudden unexpected death in epilepsy. Individuals with a confirmed heterozygous variant are indicated by +/m; individuals negative for the variant are indicated by +/+.

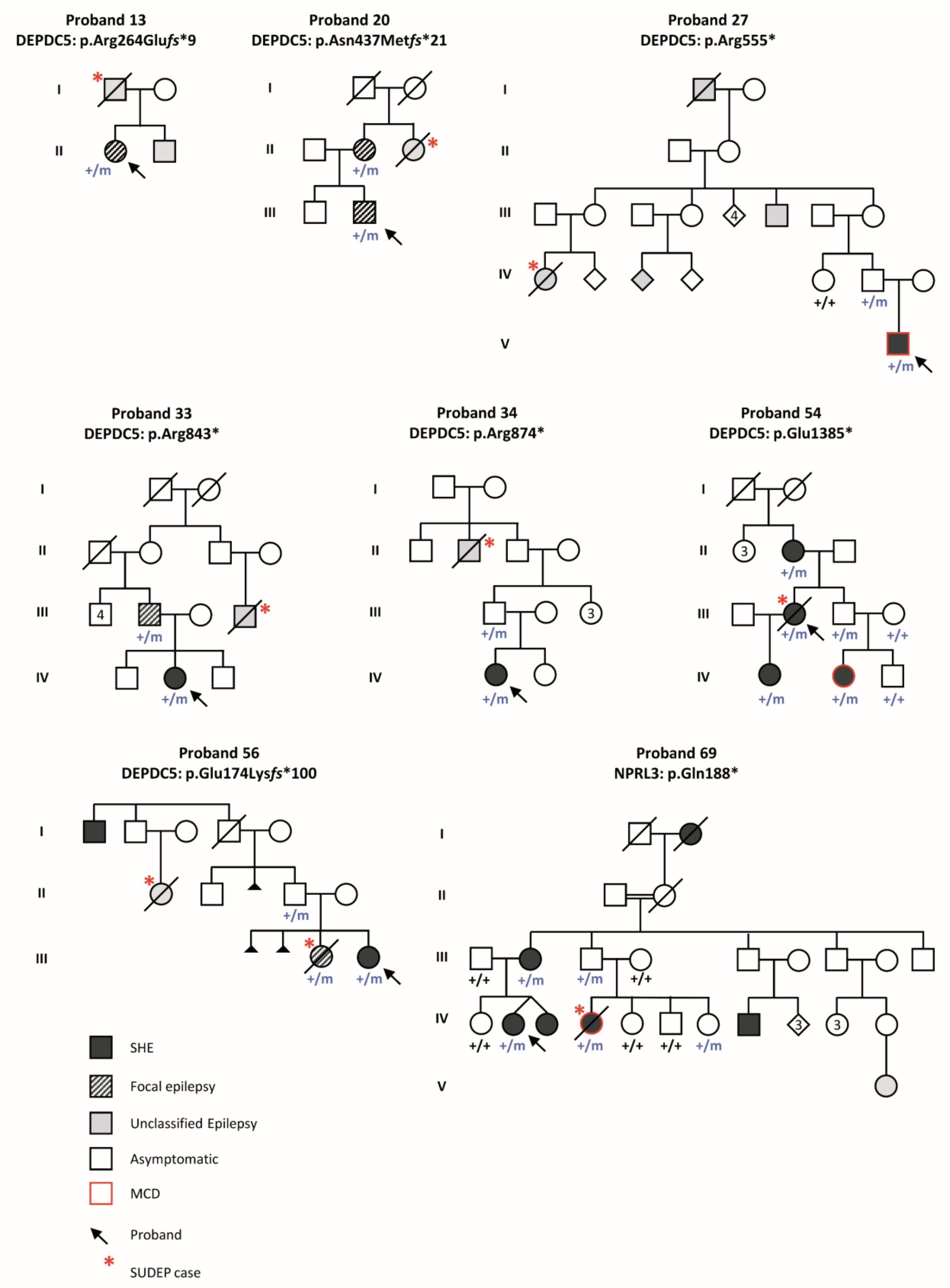

Supplement: Supplementary file 1 — Supplementary Figure S1 [file 41436_2018_60_MOESM1_ESM.pdf]
