# Supplementary material for: The landscape of epilepsy-related GATOR1 variants
Source: Genet Med. 2018 Aug 10;21(2):398–408. doi: 10.1038/s41436-018-0060-2 (PMC6292495; doi:10.1038/s41436-018-0060-2)

**Supplementary Figure S2:** Pie charts representing the distribution of the type of the 140 GATOR1 variants reported in epilepsy individuals and all GATOR1 variants described in gnomAD, except for silent variants including synonymous, intronic and UTR variants.

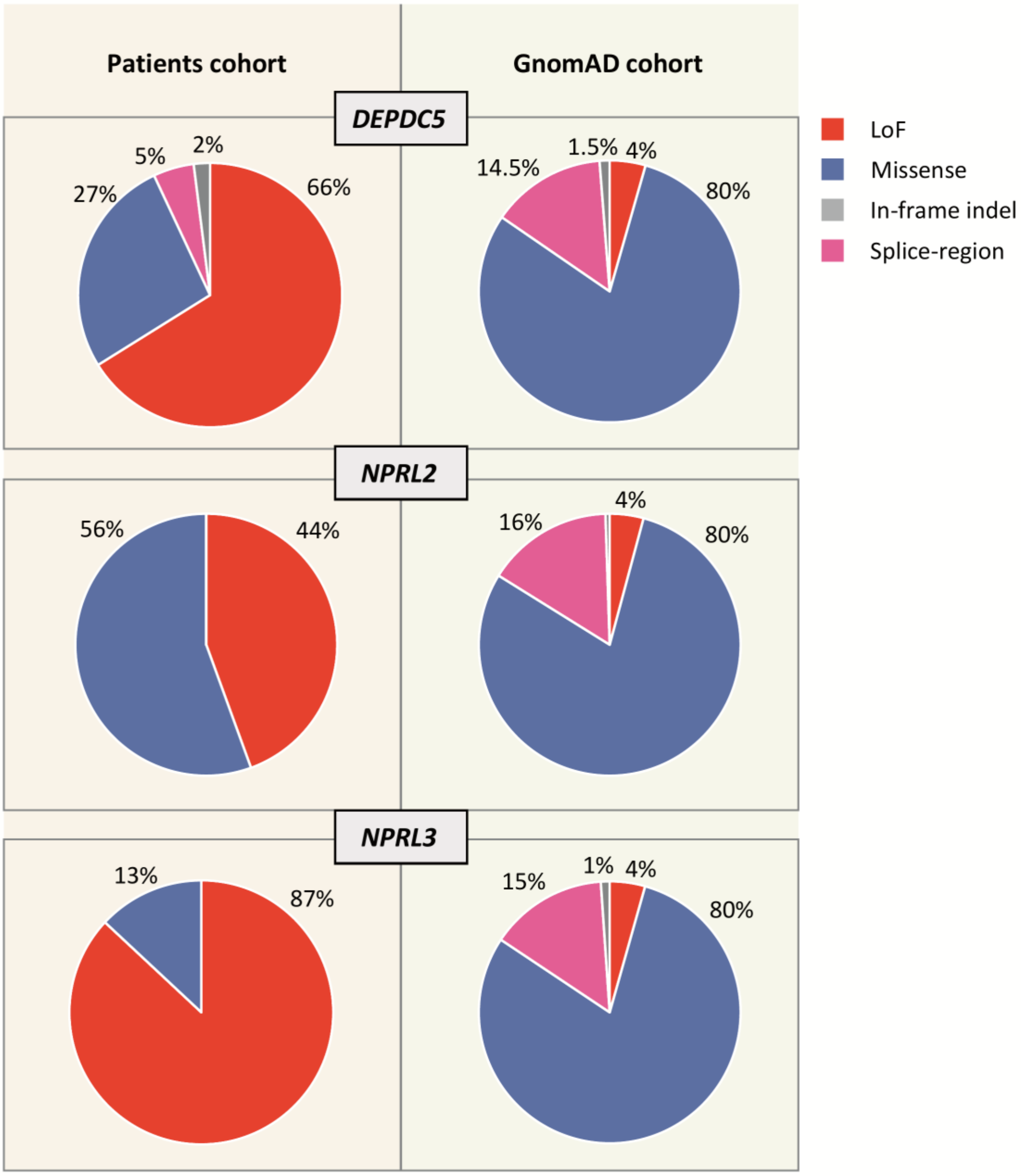

Supplement: Supplementary file 2 — Supplementary Figure S2 [file 41436_2018_60_MOESM2_ESM.pdf]
