# Supplementary material for: The landscape of epilepsy-related GATOR1 variants
Source: Genet Med. 2018 Aug 10;21(2):398–408. doi: 10.1038/s41436-018-0060-2 (PMC6292495; doi:10.1038/s41436-018-0060-2)

Supplementary Figure S3: Diagrams generated with Lollipops software showing the 38 missense variants identified in epilepsy individuals and the 1080 missense variants reported in gnomAD controls in *DEPDC5*, *NPRL2* and *NPRL3*. Variants classified as likely pathogenic are indicated in red, while variants of uncertain significance (VUS) or likely benign variants are indicated in blue. The protein domains of DEPDC5, NPRL2 and NPRL3 were adapted considering the recent cryo-electron microscopy resolution of the GATOR1 complex (Shen et al., 2018). No missense variant has been identified in the DEP domain in patients with epilepsy, and the reported missense variants did not significantly cluster in any of the four other domains of DEPDC5.

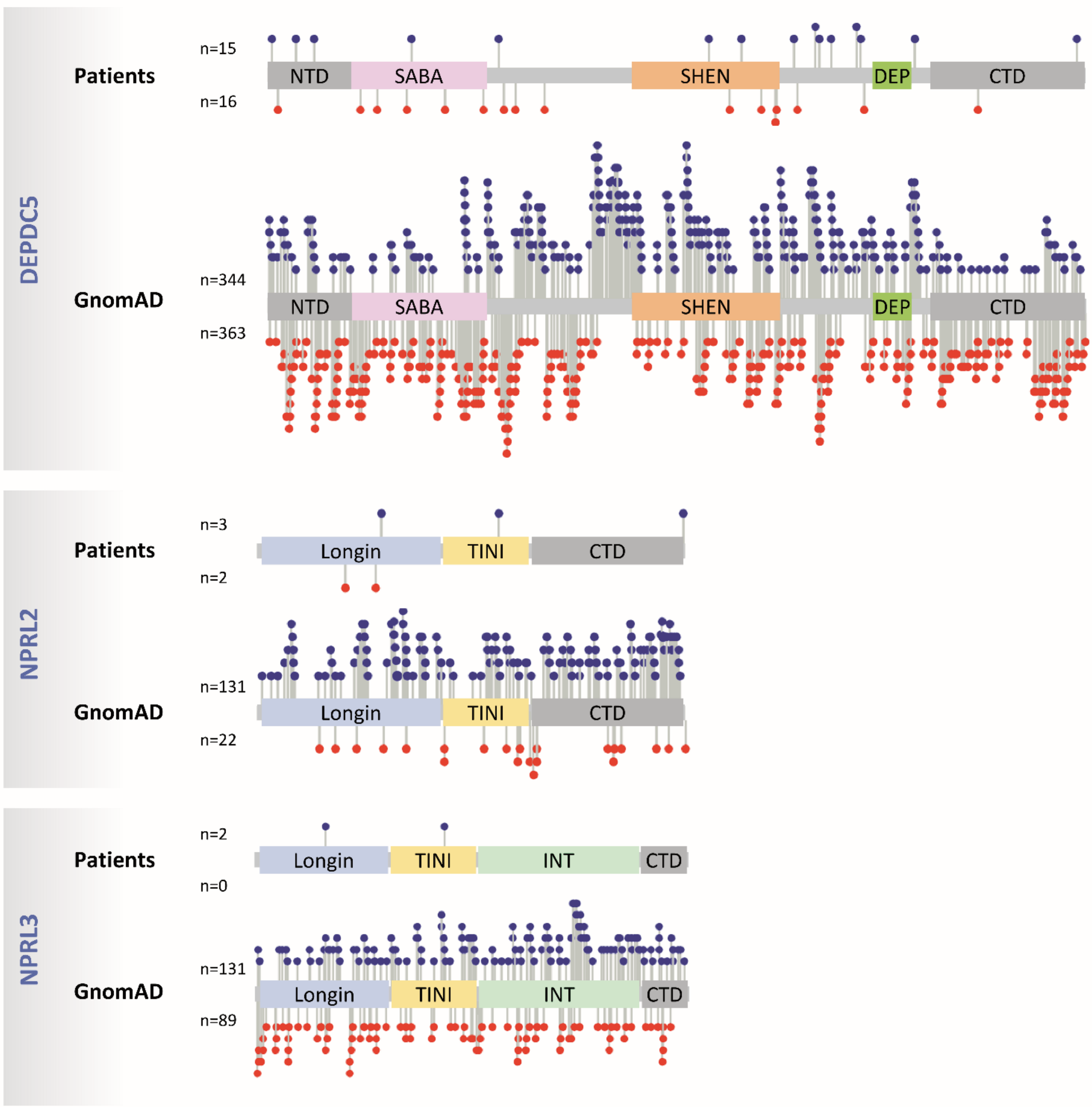

Supplement: Supplementary file 3 — Supplementary Figure S3 [file 41436_2018_60_MOESM3_ESM.pdf]
