# Supplementary material for: The landscape of epilepsy-related GATOR1 variants
Source: Genet Med. 2018 Aug 10;21(2):398–408. doi: 10.1038/s41436-018-0060-2 (PMC6292495; doi:10.1038/s41436-018-0060-2)
Supplement: Supplementary file 4 — Supplementary Information [file 41436_2018_60_MOESM4_ESM.docx]

**SUPPLEMENTARY METHODS**

**Genetic focal epilepsy global prevalence estimation.**

We estimated the prevalence of genetic focal epilepsies in the population to be 0.32% by considering a 0.76% prevalence of lifetime epilepsy (Fiest et al., 2017), among which 70% of cases have a genetic etiology (Hildebrand et al., 2013) and 60% have a focal origin (Panayiotopoulos, 2005).

- Global *genetic* epilepsy prevalence:

$$\frac{global lifetime epilepsy prevalence}{100}*70= \frac{0.76\%}{100}*70=0.53\%$$

- Global genetic *focal* epilepsy prevalence:

$$\frac{global genetic epilepsy prevalence}{100}*60= \frac{0.53\%}{100}*60=0.32\%$$

**Calculation of allele frequency threshold for classification of *likely benign* variants.**

We calculated an allele frequency threshold to classify benign variants as recently reported for *MYH7* gene (Kelly et al., 2018), considering the global prevalence of genetic focal epilepsy (0.32:100, 0.32:200 chromosomes), the contribution of *DEPDC5* (the most frequently mutated among GATOR1 encoding genes, 9.4%), and the penetrance of GATOR1 related epilepsies (60%) (Baulac, 2016). *DEPDC5* gene contribution was calculated from Ricos et al. (2016), reporting 28 *DEPDC5*-mutated patients in a cohort of 404 epilepsy patients (6.9%, CI 4.4-9.4%).

$$\frac{\left( genetic focal epilepsy prevalence \right)*\left( \% DEPDC5 contribution \right)}{\% penetrance}=\frac{\left( 0.32/200 \right)*9.4\%}{60\%}=0.00025$$

The identified allele frequency threshold would equal 69 alleles count in gnomAD (considering all 277,264 chromosomes included). This count was corrected to include the 95% of a Poisson distribution (as previously reported in Kelly et al. [2018]), leading to a maximum allele count in gnomAD of 83 (0.03% allele frequency).

**Evaluation of missense variants clustering among DEPDC5 protein domains.**

We evaluated the clustering of missense variants reported in patients and in gnomAD control cohorts in the different DEPDC5 protein domains recently described by Shen et al. (2018). 18/31 (58%) and 448/707 (63%) missense variants reported in epilepsy patients and gnomAD are located within protein domains. No significant clustering was identified.

|  |  | **Epilepsy cohort** | | | | | **GnomAD cohort** | | | | |
| --- | --- | --- | --- | --- | --- | --- | --- | --- | --- | --- | --- |
|  |  | **Obs** | **Exp** | **Obs/Exp** | **P value (≤obs)** | **P value (≥obs)** | **Obs** | **Exp** | **Obs/Exp** | **P value (≤obs)** | **P value (≥obs)** |
| **Missense variants within DEPDC5 domains** | **NTD (165 aa)** | 4 | 2.70 | 1.48 | 0.88 | 0.28 | 75 | 67.14 | 1.12 | 0.87 | 0.16 |
|  | **SABA (259 aa)** | 6 | 4.23 | 1.42 | 0.89 | 0.23 | 100 | 105.39 | 0.95 | 0.30 | 0.74 |
|  | **SHEN (290 aa)** | 6 | 4.74 | 1.27 | 0.83 | 0.33 | 126 | 118.00 | 1.07 | 0.82 | 0.21 |
|  | **DEP (75 aa)** | 0 | 1.23 | 0.00 | 0.28 | 1.00 | 33 | 30.52 | 1.08 | 0.72 | 0.35 |
|  | **CTD (312 aa)** | 2 | 5.10 | 0.39 | 0.08 | 0.98 | 114 | 126.95 | 0.90 | 0.09 | 0.92 |

*Binomial one-sided test, 95% confidence interval. Obs: number of missense variants observed; Exp: number of missense variants expected considering a uniform distribution of the observed missense variants along the DEPDC5 protein domains. aa: amino acid length of the domain.*

The same analysis could not be performed for NPRL2 or NPRL3 proteins because the reported number of missense variants in epilepsy patients is too low for a statistical analysis.

**SUPPLEMENTARY DATA LEGENDS**

**Supplementary Figure S1:** Pedigrees of GATOR1 families with SUDEP reported cases. SHE: sleep-related hypermotor epilepsy; MCD: malformation of cortical development; SUDEP: sudden unexpected death in epilepsy. Individuals with a confirmed heterozygous variant are indicated by +/m; individuals negative for the variant are indicated by +/+.

**Supplementary Figure S2:** Pie charts representing the distribution of the type of the 140 GATOR1 variants reported in epilepsy individuals and all GATOR1 variants described in gnomAD, except for silent variants (including synonymous, intronic and UTR variants).

**Supplementary Figure S3**: Diagrams generated with Lollipops software showing the 38 missense variants identified in epilepsy individuals and the 1080 missense variants reported in gnomAD controls in *DEPDC5*, *NPRL2* and *NPRL3*. Variants classified as likely pathogenic are indicated in red, while variants of uncertain significance (VUS) or likely benign variants are indicated in blue. The protein domains of DEPDC5, NPRL2 and NPRL3 were adapted considering the recent cryo-electron microscopy resolution of the GATOR1 complex (Shen et al., 2018). No missense variant has been identified in the DEP domain in patients with epilepsy, and the reported missense variants did not significantly cluster in any of the four other domains of DEPDC5.

**Supplementary Table S1**: Detailed clinical features of the 73 epilepsy probands included in this study and 26 affected family members for which clinical information were available. The data are divided in two separate sheets for probands and affected relatives. ID: intellectual disability; ASD: autism spectrum disorder; ADHD: attention deficit hyperactivity disorder; FCD: focal cortical dysplasia; N/A: not available. ACZ: acetazolamide; BZD: benzodiazepine; CBZ: carbamazepine; CLB: clobazam; CZP: clonazepam; ESL: eslicarbazepine; ETX: ethosuximide; FBM: felbamate; GBP: gabapentine; LCS: lacosamide; LEV: levetiracetam; LTG: lamotrigine; OXC: oxcarbazepine; PB: phenobarbital; PER: perampanel; PGB: pregabalin; PHT: phenytoin; PRM: primidone; PSL: prednisolone; RFM: rufinamide; STM: sulthiame; TPM: topiramate; VGB: vigabatrin; VNS: vagal nerve stimulation; VPA: valproate; ZSM: zonisamide.

**Supplementary Table S2**: Full list of the variants and main clinical information of 183 unrelated families with GATOR1 variants reported so far.

**SUPPLEMENTARY REFERENCES**

**Supplementary methods**

Fiest KM, Sauro KM, Wiebe S, et al. Prevalence and incidence of epilepsy: A systematic review and meta-analysis of international studies. *Neurology.* 2017;88(3):296-303.

Hildebrand MS, Dahl HH, Damiano JA, Smith RJ, Scheffer IE, Berkovic SF. Recent advances in the molecular genetics of epilepsy. *Journal of medical genetics.* 2013;50(5):271-279.

Panayiotopoulos CP. Syndromes of idiopathic generalized epilepsies not recognized by the International League Against Epilepsy. *Epilepsia.* 2005;46 Suppl 9:57-66.

Kelly MA, Caleshu C, Morales A, et al. Adaptation and validation of the ACMG/AMP variant classification framework for MYH7-associated inherited cardiomyopathies: recommendations by ClinGen's Inherited Cardiomyopathy Expert Panel. *Genet Med.* 2018.

Baulac S. mTOR signaling pathway genes in focal epilepsies. *Progress in brain research.* 2016;226:61-79.

Ricos MG, Hodgson BL, Pippucci T, et al. Mutations in the mammalian target of rapamycin pathway regulators NPRL2 and NPRL3 cause focal epilepsy. *Ann Neurol.* 2016;79(1):120-131.

Shen K, Huang RK, Brignole EJ, et al. Architecture of the human GATOR1 and GATOR1-Rag GTPases complexes. *Nature.* 2018; 556(7699):64-69.

**Supplementary Table S2**

Bagnall RD, Crompton DE, Petrovski S, et al. Exome-based analysis of cardiac arrhythmia, respiratory control, and epilepsy genes in sudden unexpected death in epilepsy. *Ann Neurol.* 2016;79(4):522-534.

Baulac S, Ishida S, Marsan E, et al. Familial focal epilepsy with focal cortical dysplasia due to DEPDC5 mutations. *Ann Neurol.* 2015;77(4):675-683.

Bisulli F, Licchetta L, Baldassari S, Pippucci T, Tinuper P. DEPDC5 mutations in epilepsy with auditory features. *Epilepsia.* 2016;57(2):335.

Bonaglia MC, Giorda R, Epifanio R, et al. Partial deletion of DEPDC5 in a child with focal epilepsy. *Epilepsia Open.* 2017;1(3-4):140-144.

Carvill GL, Crompton DE, Regan BM, et al. Epileptic spasms are a feature of DEPDC5 mTORopathy. *Neurol Genet.* 2015;1(2):e17.

Cen Z, Guo Y, Lou Y, Jiang B, Wang J, Feng J. De novo mutation in DEPDC5 associated with unilateral pachygyria and intractable epilepsy. *Seizure : the journal of the British Epilepsy Association.* 2017;50:1-3.

D'Gama AM, Geng Y, Couto JA, et al. Mammalian target of rapamycin pathway mutations cause hemimegalencephaly and focal cortical dysplasia. *Ann Neurol.* 2015;77(4):720-725.

Dibbens LM, de Vries B, Donatello S, et al. Mutations in DEPDC5 cause familial focal epilepsy with variable foci. *Nature genetics.* 2013;45(5):546-551.

Ishida S, Picard F, Rudolf G, et al. Mutations of DEPDC5 cause autosomal dominant focal epilepsies. *Nature genetics.* 2013;45(5):552-555.

Korenke GC, Eggert M, Thiele H, Nurnberg P, Sander T, Steinlein OK. Nocturnal frontal lobe epilepsy caused by a mutation in the GATOR1 complex gene NPRL3. *Epilepsia.* 2016;57(3):e60-63.

Lal D, Reinthaler EM, Schubert J, et al. DEPDC5 mutations in genetic focal epilepsies of childhood. *Ann Neurol.* 2014;75(5):788-792.

Martin C, Meloche C, Rioux MF, et al. A recurrent mutation in DEPDC5 predisposes to focal epilepsies in the French-Canadian population. *Clinical genetics.* 2014;86(6):570-574.

Mirzaa GM, Campbell CD, Solovieff N, et al. Association of MTOR Mutations With Developmental Brain Disorders, Including Megalencephaly, Focal Cortical Dysplasia, and Pigmentary Mosaicism. *JAMA Neurol.* 2016;73(7):836-845.

Nascimento FA, Borlot F, Cossette P, Minassian BA, Andrade DM. Two definite cases of sudden unexpected death in epilepsy in a family with a DEPDC5 mutation. *Neurol Genet.* 2015;1(4):e28.

Perucca P, Scheffer IE, Harvey AS, et al. Real-world utility of whole exome sequencing with targeted gene analysis for focal epilepsy. *Epilepsy research.* 2017;131:1-8.

Picard F, Makrythanasis P, Navarro V, et al. DEPDC5 mutations in families presenting as autosomal dominant nocturnal frontal lobe epilepsy. *Neurology.* 2014;82(23):2101-2106.

Pippucci T, Licchetta L, Baldassari S, et al. Epilepsy with auditory features: A heterogeneous clinico-molecular disease. *Neurol Genet.* 2015;1(1):e5.

Ricos MG, Hodgson BL, Pippucci T, et al. Mutations in the mammalian target of rapamycin pathway regulators NPRL2 and NPRL3 cause focal epilepsy. *Ann Neurol.* 2016;79(1):120-131.

Scerri T, Riseley JR, Gillies G, et al. Familial cortical dysplasia type IIA caused by a germline mutation in DEPDC5. *Annals of clinical and translational neurology.* 2015;2(5):575-580.

Scheffer IE, Heron SE, Regan BM, et al. Mutations in mammalian target of rapamycin regulator DEPDC5 cause focal epilepsy with brain malformations. *Ann Neurol.* 2014;75(5):782-787.

Sim JC, Scerri T, Fanjul-Fernandez M, et al. Familial cortical dysplasia caused by mutation in the mammalian target of rapamycin regulator NPRL3. *Ann Neurol.* 2016;79(1):132-137.

Striano P, Serioli E, Santulli L, et al. DEPDC5 mutations are not a frequent cause of familial temporal lobe epilepsy. *Epilepsia.* 2015;56(10):e168-171.

Tsai MH, Chan CK, Chang YC, et al. DEPDC5 mutations in familial and sporadic focal epilepsy. *Clinical genetics.* 2017;92(4):397-404.

Weckhuysen S, Marsan E, Lambrecq V, et al. Involvement of GATOR complex genes in familial focal epilepsies and focal cortical dysplasia. *Epilepsia.* 2016;57(6):994-1003.

**Supplementary Figure S3**

Jay JJ, Brouwer C. Lollipops in the Clinic: Information Dense Mutation Plots for Precision Medicine. *PLoS One*. 2016;11(8):e0160519.

Shen K, Huang RK, Brignole EJ, et al. Architecture of the human GATOR1 and GATOR1-Rag GTPases complexes. *Nature.* 2018; 556(7699):64-69.
